# Differences for traits associated with early N acquisition in a grain legume and early complementarity in grain legume–triticale mixtures

**DOI:** 10.1093/aobpla/ply001

**Published:** 2018-01-04

**Authors:** Nicolas Carton, Christophe Naudin, Guillaume Piva, Rim Baccar, Guénaëlle Corre-Hellou

**Affiliations:** USC LEVA, INRA, Ecole Supérieure d’Agricultures, Univ. Bretagne Loire, Angers Cedex, France

**Keywords:** Cereal–legume intercropping, crop competitive ability, early growth, *Lupinus*, mineral soil N uptake, root traits

## Abstract

Early strategies of crop growth and N acquisition can be critical for determining competitive interactions between weeds and crops. Grain legumes and especially lupins are known to be poor competitors against weeds. Grain legumes are known to have low mineral soil N uptake abilities. However, inter- and intraspecific differences in N uptake ability in relation to below-ground traits have received little attention. Our objectives were (i) to measure differences among lupins for a set of traits associated with early growth and N acquisition; (ii) to examine how this variation compares to differences between lupin and a cereal, triticale, and (iii) to assess if mixing lupin with triticale provides a higher potential than does pure lupin regarding plant biomass and mineral soil N acquisition early in the crop cycle. Lupin (12 genotypes) and triticale plants were grown separately and in mixed species pairs in a replacement design for 1 and 2 months in three rhizotron experiments. Shoot and root biomass, root length, root expansion dynamics, N_2_ fixation and mineral soil N uptake were measured. Differences among lupin species and genotypes regarding traits related to early growth and to mineral soil N uptake were observed, but all lupins demonstrated slow early growth and low ability to absorb mineral soil N compared to triticale. In lupin–triticale mixture, a contrast in early growth strategies between species induced a higher total soil mineral N uptake compared with pure lupin. Complementarity between lupin and triticale persisted during the second month, when interactions began. This complementarity may allow for reduced competition between species, favouring higher triticale biomass production than in pure triticale, without compromising lupin growth.

## Teaser

We grew a grain legume in rhizotrons, alone and mixed with a cereal. There were differences between some of the genotypes of the grain legume but they all demonstrated slow early growth and low ability to absorb mineral soil nitrogen compared to the cereal. The two species were complementary for nitrogen acquisition in the first 2 months of growth. Mixtures showed a higher total soil mineral nitrogen uptake than pure grain legume. Complementarity allowed higher cereal biomass production than in pure cereal, without compromising grain legume growth.

## Introduction

To reduce dependency on chemical weed control, managing crop competitive ability is a key component of integrated weed management (Jordan 1993). In particular, competitive ability depends on above- and below-ground organs and their ability to absorb light, water and nutrients. In return, plant organ growth responds to the amount of resources absorbed. Plant functional traits can affect their competitive effect and response and are thus relevant tools for studying competitive ability (Pakeman *et al.* 2015; Schwartz *et al.* 2016). Most crop competitive ability studies have explored above-ground traits and have identified key traits involved in crop–weed competition such as height, leaf area and branch number (e.g. Christensen 1995; Lemerle *et al.* 1996; Bussan *et al.* 1997; Jacob *et al.* 2016). Nevertheless, such studies have not identified systematically strong correlations between weed suppression and above-ground traits, suggesting that below-ground traits may play a significant role. Crop below-ground competitive ability has been less studied than the above-ground ability despite evidence that competition between plants for water and nutrients is as strong or stronger than competition for light (Martin and Snaydon 1982; Satorre and Snaydon 1992; Casper and Jackson 1997), especially at low nutrient levels (Kiær *et al.* 2013). Rapid early growth is considered crucial in crop–weed competition (Lemerle *et al.* 2001) as small advantages in early season seedling growth can translate into large differences in size and ability to capture resources later in the season (Fukai and Trenbath 1993). However, few studies have examined above- and below-ground traits during initial growth stages (Thorup-Kristensen 1998).

The ability of plants to absorb below-ground resources is linked to traits related to soil volume exploration (root density or root length density, branch spacing) and root dynamics (relative growth rate; Robinson *et al.* 1999; Thorup-Kristensen 2001; Cahill 2003; Dunbabin *et al.* 2003; Dunbabin 2007; Pierik *et al.* 2013). Early competitive ability for nitrogen (N) is also related to plant N demand, i.e. early growth rate and the extent to which seed reserves can fulfil plant N needs (seed DW (dry weight) and N content).

Legumes appear to be generally less competitive than non-N-fixing plants for mineral soil N uptake. Hauggaard-Nielsen *et al.* (2001) suggested that the stronger weed growth often observed in legume crops in comparison to non-N-fixing crops is a consequence of high mineral soil N availability due to the low soil N acquisition by the crop. During early growth, legumes have access to three sources of N: seed N, mineral soil N and atmospheric N_2_. During the first stages of development, legumes rely mainly on mineral soil N uptake for exogenous N like non-legume species (Dayoub *et al.* 2017), as the contribution of N_2_ fixation to N demand is low due to the delay needed for the establishment of nodules. N_2_ fixation has been reported in measurable amounts in several legume species 35 days after germination at 20 °C (Dayoub *et al.* 2017) and as soon as 240 degree-days in pea (Voisin *et al.* 2002). Grain legumes can also rely on seed N reserves for a significant period of time because their large, N-rich seeds lead to the low use of external N sources (Herdina and Silsbury 1990; Dayoub *et al.* 2017). Shallow root depth, low root length density (Hamblin and Tennant 1987) and slow root growth partly due to the energy cost for the construction of N-fixing structures (Warembourg 1983) can also contribute to the low soil mineral N uptake ability of grain legumes.

White lupin (*Lupinus albus*), yellow lupin (*L. luteus*) and blue lupin (*L. angustifolius*) are native Mediterranean legumes with a high seed protein concentration close to that of soybean (Lucas *et al.* 2015). Lupins are considered a significant alternative to soybean imports for feed that also can be used in food. Moreover, like other grain legumes, lupins produce protein-rich grains with a reduced environmental impact due to symbiotic N_2_ fixation, which allows for improved N fertilizer economy in cropping systems (Crews and Peoples 2004). However, lupins are known to be very poor competitors against weeds (e.g. Lemerle *et al.* 1995; Kruidhof *et al.* 2008), which is believed to contribute to their highly variable yield that has limited their adoption in cropping systems.

Interspecific (Dayoub *et al.* 2017) and intraspecific variation for traits related to soil volume exploration and plant N needs have been observed among legumes, in pea (McPhee 2005) and in blue lupin (Chen *et al.* 2012). However, in the genus *Lupinus*, no study has specifically assessed interspecific variation among lupin species and intraspecific differences among white lupin genotypes for traits related to N uptake ability.

Although there may be differences among lupins, traits may be too similar to significantly increase early competitive ability. Intercropping is another method to further increase trait diversity that may yield superior results. By replacing some lupin plants with plants of another crop species that have complementary traits, early competitive ability of the crop against weeds could be more efficiently increased. Cereals have a stronger ability to absorb soil mineral N than do grain legumes (Jensen 1996; Hauggaard-Nielsen *et al.* 2001; Bellostas *et al.* 2003; Corre-Hellou *et al.* 2007), and N use was assumed to be the most important factor for improved weed suppression in pea–barley intercrops compared to pea pure crops under low mineral soil N (Corre-Hellou *et al.* 2011). Thus, intercropping with a non-N-fixing plant that has a high N demand may be of particular interest for legume crops that lack strong competitive ability for mineral soil N. Moreover, when intercropping a legume with a non-N-fixing crop, the use of two N sources (mineral soil N and N_2_) generally leads to stronger growth and higher overall crop N content. Non-N-fixing plants usually benefit from reduced competition in mixed stands compared to pure stands and attain higher biomass and N content per plant (Corre-Hellou *et al.* 2006). It has also been shown that species can exhibit plasticity in their traits associated with N uptake in response to intercropping (Liu *et al.* 2015). Early growth is of key importance in the onset and subsequent dynamics of interactions in intercrops (Tofinga *et al.* 1993; Andersen *et al.* 2007; Corre-Hellou *et al.* 2007; Naudin *et al.* 2010; Fayaud *et al.* 2014). Several studies have shown that cereals outcompete legumes in cereal–legume intercrops, but the differences in N acquisition strategies in relation to root traits and seed characteristics during the early growth phase between legumes and cereals have not been studied.

The objectives of the present study were (i) to measure the range of variation between lupin genotypes and species for a set of traits with respect to early growth and N acquisition, (ii) to examine how this variation in traits compares to differences between lupin and a cereal, triticale, and (iii) to assess if mixing lupin with triticale provides a higher potential for plant biomass and the acquisition of mineral soil N early in the crop cycle compared with pure lupin. To achieve these objectives, three rhizotron experiments were conducted in controlled conditions involving lupin and triticale plants grown both separately and in mixed species pairs, and a set of traits (biomass, plant N content and origin, root system characteristics) were measured during early growth.

## Methods

### Plant material and experimental design

Three species of lupin were studied during early growth stages: white lupin (*L. albus*), blue lupin (*L. angustifolius*) and yellow lupin (*L. luteus*). Eight winter white lupin genotypes and varieties were selected based on their contrasting traits related to seed size and early growth (Table 1). Winter white lupin genotypes were selected from a French winter white lupin selection programme (Jouffray-Drillaud). The lupin species and genotypes were compared to triticale (×*Triticosecale*).

**Table 1.** Genotypes used in the three experiments. WWL: winter white lupin, *Lupinus albus*; SWL: spring white lupin, *Lupinus albus*; BL: blue lupin, *Lupinus angustifolius*; YL: yellow lupin, *Lupinus luteus*; T: triticale, ×*Triticosecale*.

Code	WWL-1	WWL-2	WWL-3	WWL-4	WWL-5	WWL-6	WWL-7	WWL-8	SWL-1	SWL-2	BL	YL	T-1	T-2
Name	889b13	714a3-3	Clovis	Luxe	598c2	Magnus	441a21-31	871j4	Feodora	Energy	Arabella	Mister	Ragtac	Vuka
Present in Expt.	1, 2	1	1	1	1	1, 3	1	1, 2	1, 2	1, 2	1	1	1	2, 3

Three experiments were conducted in a greenhouse under natural light in western France (47°28′0″N, 0°47′31″W) in 2015 and 2016 (Table 2). Plants were grown in transparent rhizotrons (Jamont *et al.* 2013). All experiments were carried out with low mineral soil N availability using five replicates per experimental unit, except in rare cases of seedling death (three or four replicates).

**Table 2.** Description of methods of the three rhizotron experiments. WWL: winter white lupin; SWL: spring white lupin; BL: blue lupin; YL: yellow lupin; T: triticale.

Experiment	Dates (sowing to harvest)	Duration (days)	Rhizotron size (cm)	Plant species	Plants per rhizotron	Mean temperature (°C)	Initial soil mineral N content (mg kg^−1^)
Expt. 1	22/01/15 to 24/02/15	33	50 × 20 × 3	WWL (8), SWL (2), BL (1), YL (1), T (1)	1	13.7	8.43
Expt. 2	18/01/16 to 17/02/16	30	50 × 20 × 3	WWL (2), SWL (2), T (1)	2	14.2	11.44
Expt. 3	04/04/16 to 01/06/16	58	60 × 25 × 5	WL (1), T (1)	2	17.8	3.62 + 5 mg supplied though irrigation water

Expt. 1 aimed at exploring differences in early growth traits at different levels: intra-species (10 white lupin genotypes), inter-species within the genus *Lupinus* (winter white lupin (WWL), spring white lupin (SWL), blue lupin (BL) and yellow lupin (YL)) and inter-family (white lupins and triticale). Expt. 2 and Expt. 3 aimed at assessing if lupin–triticale mixture has a higher plant biomass and N acquisition than does pure lupin after 1 (Expt. 2) and 2 months (Expt. 3). In Expt. 1, one plant of each genotype was grown per rhizotron. In Expt. 2 and Expt. 3, two plants were grown in each rhizotron in a replacement design. Pure lupin (two lupin plants), lupin mixed with triticale (one lupin plant and one triticale plant) and pure triticale (two triticale plants) were studied with four white lupin genotypes in Expt. 2 and with one winter white lupin variety in Expt. 3.

The rhizotrons (inner dimensions in Table 2) were covered with black polyethylene plastic sheeting to avoid exposing the roots to light and inclined at 45° to force the roots to grow along the underside. Each rhizotron was filled with a mixture of sandy soil (62.3 % sand, 24.1 % silt, 11.5 % clay) equal to 25 % of the total dry mass and with dry sand equal to 75 % of total dry mass. The sand was previously washed and dried at 105 °C for 48 h, and the soil was sieved (7 mm) before homogeneous soil–sand mixing. Soil mineral N availability in the final mixed soil was lower than 12 mg kg^−1^ in the three experiments (Table 2). The final composition of the soil–sand mixture was 2.9 % clay, 6 % loam, 90.6 % sand and 0.4 % organic matter, and the pH of the mixture was 6.9. The phosphorus (P Olsen) and potassium (K) concentrations were 30.3 and 78.7 mg kg^−1^ of dry soil, respectively. An N-free nutrient solution was added to each rhizotron at the following concentrations (mol L^−1^): 7.3 × 10^–4^ KCl, 2.3 × 10^–4^ KH_2_PO_4_·7H_2_O, 1.6 × 10^–4^ MgSO_4_, 1.2 × 10^–4^ CaCl_2_ and 2.5 × 10^–5^ FeCl_3_. The nutrient solution was mixed with the soil before filling the rhizotrons. One hundred and fifty millilitres of this solution were used in Expt. 1 and Expt. 2 and 418 mL in Expt. 3.

The seeds were weighed and calibrated to reduce variation in seed mass within genotypes to less than ± 5 %. All seeds were treated for 10 min in a solution containing 0.317 % NaClO (surface sterilization) and thoroughly rinsed, after which they were germinated on filter paper until the radicle emerged from the seed (BBCH stage 0.5; Lancashire *et al.* 1991). In Expt. 1, the seed was placed in the middle of the rhizotron surface, and in Expt. 2 and Expt. 3, the seeds were placed so that they would be equidistant from the rhizotron edge and the other seed. Sowing depth was 1.0 cm for lupin and 0.5 cm for triticale. All seeds were then inoculated with *Bradyrhizobium lupini* at an estimated rate of 10^8^ cells per plant to allow for N_2_ fixation in lupins (Naudin *et al.* 2011). After seedling emergence, a 1-cm-thick layer of sand was added to the surface of the rhizotron to limit water evaporation from the soil. In Expt. 1 and Exp. 2, the rhizotron soil was maintained in a water content range slightly below field capacity with a total quantity of 30 and 100 mL, respectively. Demineralized water was added as needed, and in Expt. 3, due to the longer growth period and higher temperature, the rhizotrons were supplemented with tap water to maintain soil in the desired water content range (2.0 L per rhizotron during the experiment). Control rhizotrons without plants were included in Expt. 1 and Expt. 2 (three replicates) to measure mineral soil N availability changes during the experiment.

Three lupin plants for each studied genotype were also grown simultaneously in N-free sand to determine the ^15^N abundance of plants relying only on N_2_ fixation (β-value − isotopic fractionation factor of each genotype of lupin) to help estimate the N_2_ fixation according to the natural abundance method (Amarger *et al.* 1979). The pots in Expt. 1 and Expt. 2 were supplied with 80 mL of a N-free solution containing (mol L^−1^) 5.4 × 10^–4^ KCl, 1.7 × 10^–4^ KH_2_PO_4_, 1.2 × 10^–4^ MgSO_4_·7H_2_O, 9 × 10^–5^ CaCl_2_ and 1.8 × 10^–5^ FeCl_3_ and in Expt. 3 with 60 mL of a N-free nutrient solution containing (mol L^−1^) 1.1 × 10^–3^ KCl, 3.4 × 10^–4^ KH_2_PO_4_, 2.4 × 10^–4^ MgSO_4_·7H_2_O, 1.8 × 10^–4^ CaCl_2_ and 3.7 × 10^–5^ FeCl_3_ at sowing and once per week. Micronutrients were supplied in the nutrient solutions at the following concentrations (mol L^−1^): 2 × 10^–4^ MnSO_4_·H_2_O, 2 × 10^–4^ H_3_BO_3_, 6 × 10^–6^ ZnSO_4_·7H_2_O, 1 × 10^–6^ Na_2_Mo_4_·2H_2_O, 2 × 10^–7^ CuSO_4_·5H_2_O and 2 × 10^–7^ CoCl_2_·6H_2_O. The pots were maintained in the desired water content range by adding demineralized water as needed.

### Measurements and harvest

In Expt. 1 and Expt. 2, rooting depth was monitored by drawing visible roots three times a week on a transparent plastic sheet fixed to the underside of the rhizotron. In Expt. 1, root density was monitored using a grid of 1-cm × 1-cm squares and counting the number of squares containing a root on each monitoring date. Root density data for triticale are not presented because it appeared that only a small proportion of triticale roots were visible on the rhizotron. Visible triticale roots were, however, considered sufficient to monitor triticale rooting depth.

In Expt. 1 and Expt. 2, the plants were harvested while still in the early growth phase (33 days in Expt. 1 and 30 days in Expt. 2). At harvest, growth stages were BBCH 1.3 to 1.7 for lupins and 2.1 for triticale. In Expt. 3, lupin was harvested at growth stage 1.18 (18 leaves, before stem elongation), and triticale was harvested at growth stage 3.3 (Lancashire *et al.* 1991).

At harvest, root systems were removed from the rhizotron and washed with demineralized water. In Expt. 1 and Expt. 2, root systems were then stored at 4.0 °C in a solution of 0.5 mM CaCl_2_ for a maximum of 2 h before further manipulation. Nodules were separated from the roots and counted. In Expt. 1 and Expt. 2, the root systems were spread on a glass recording tray and scanned at 600 dpi in grey scale with an Epson Perfection V700 Scanner with a transparency scanning system (Seiko Epson Corporation, Suwa, Nagano, Japan), and the root length was measured using WinRHIZO software (WinRHIZO Pro2012b; Regent Instruments Inc., Quebec, Canada).

Plants grown in N-free sand (helping to estimate the respective β-value) were harvested at the same date. After oven-drying (48 h at 70 °C), the dry matter of roots, shoots and nodules were determined. All of the samples (including calibrated seeds) were ground to a fine powder for total N (Dumas procedure), and all ^15^N:^14^N ratio measurements of the original seeds, roots + nodules and shoots were performed using a CHN analyser (EA3000; Euro Vector, Milan, Italy) and a mass spectrometer (IsoPrime; Elementar, Hanau, Germany).

At sowing and harvest, soil samples were collected, and the soil inorganic N concentration was measured using a segmented flow analysis method (SA3000; Skalar Analytical B.V., Breda, Netherlands), which enables determination of the nitrate content by KCl extraction according to the international standard ISO 14256-2.

### Calculations and statistics

Exogenous N was calculated as the difference between total plant N and seed N. The relative contributions of the exogenous sources of N (air and soil) were then determined. The proportion of plant exogenous N derived from the air (%Ndfa) was estimated using the ^15^N natural abundance method (Amarger *et al.* 1979). Triticale was used as the non-N-fixing reference plant in Equation (1) as follows:

$$\%\text{Ndfa}=\text{1}00\;\times \;[(\delta^{\text{15}}{\text{N}}_{\text{triticale}}-\delta^{\text{15}}{\text{N}}_{\text{lupin}})/(\delta^{\text{15}}{\text{N}}_{\text{lupin}}-\beta )]$$ (1)

where *δ*^15^N_lupin_ and *δ*^15^N_triticale_ express the observed natural ^15^N abundances of lupin and of triticale, respectively, and β is the isotopic fractionation factor measured from plants grown in N-free sand.

The values of *δ*^15^N for lupins and triticale were corrected to take seed N into account (Jensen *et al.* 1985) according to Equation (2) as follows:

$$\delta^{\text{15}}{\text{N}}_{\text{corrected}}=\text{ [}\delta^{\text{15}}{\text{N}}_{\text{plant}}-(({\text{QN}}_{\text{seed}}/{\text{QN}}_{\text{plant}})\times \delta^{\text{15}}{\text{N}}_{\text{seed}})\text{]/[1}-({\text{QN}}_{\text{seed}}/{\text{QN}}_{\text{plant}})]$$ (2)

where *δ*^15^N_seed_ expresses the natural ^15^N abundance of the original seeds. The contribution of mineral soil N was calculated as the difference between exogenous N and N derived from the air, assuming N derived from the air was 0 when no N_2_ fixation could be detected.

In this study, the relative competitive ability of the components in mixed stands was expressed using the ‘competitive balance index’ (Cb; Equation (3); Wilson 1988) as follows:

$$\text{Cb}={\text{ln [(Y}}_{\text{LT}}/{\text{Y}}_{\text{LL}}{\text{)/(Y}}_{\text{TL}}/{\text{Y}}_{\text{TT}}\text{)]}$$ (3)

where Y is the DW or other variables per plant; L is lupin; T is triticale; Y_LL_ is the value for pure lupin; Y_TT_ is the value of pure triticale; Y_LT_ is the value of lupin when grown with triticale; and Y_TL_ is the value of triticale when grown with lupin. Cb is close to 0 when there is no competition or when plants have the same competitive abilities; Cb is close to −1 when triticale has a higher competitive ability than lupin or benefits more from being mixed than lupin; and Cb is close to 1 when the opposite is true. The complementarity in resource use is expressed by the relative yield total (RYT) (Equation (4)) (de Wit and van den Bergh 1965) as follows:

$$\text{RYT}={\text{Y}}_{\text{LT}}/{\text{Y}}_{\text{LL}}+{\text{Y}}_{\text{TL}}/{\text{Y}}_{\text{TT}}$$ (4)

A RYT value higher than 1 indicates an advantage of mixed treatments over pure treatments.

The differences between genotypes of lupin, between white lupins and triticale and between pure and mixed stands for traits, growth parameters and performances were assessed by a one-way ANOVA (type II sum of squares; *α* = 0.05). The normality and homoscedasticity of model residues were tested using Shapiro’s and Bartlett’s tests, respectively (*α* = 0.05). In Expt. 2, the effects of neighbouring plants and lupin genotype were tested by a two-way ANOVA (type III sum of squares; *α* = 0.05). For variables where model residuals did not show normality, the data were transformed using the best function as determined by the Box–Cox procedure (Box and Cox 1964). The means were compared using Tukey’s HSD test (honest significant difference; *α* = 0.05) to determine which treatments were different. For variables where model residuals did not show homoscedasticity, Welch’s correction was applied when conducting the ANOVA (Welch 1947), and the Tukey–Games–Howell test was used to determine which treatments were different (Games and Howell 1976). We used linear regressions to assess correlations between variables in Expt. 1 as well as to calculate root vertical expansion rates (sowing to 25 days after sowing (DAS)) and root density expansion rates in the linear phase (18–25 DAS). At 25 DAS, the roots of some individuals reached the bottom of their rhizotron.

All statistical analyses were performed using R software, version 3.1.2 (R Core 2014).

## Results

### Inter- and intraspecific variation among lupin genotypes for early growth and N acquisition

#### Amount of variation for all studied traits.

Among white lupins, seed mass varied between 208 and 308 mg per seed and had a coefficient of variation (CV) of 11 % (Table 3). Seed N content showed a higher variation (CV = 23 %) and ranged from 9.9 to 22.1 mg per seed. Blue lupin and yellow lupin had lighter seeds and a lower seed N content compared with white lupin. We measured statistically significant differences among lupin genotypes for every trait related to above- and below-ground growth and root exploration (Table 3). The highest CVs were observed for root length (46 %) and nodule DW (50 %). The root vertical expansion rate and root density differed also largely among lupin genotypes (CV = 21 and 34 %, respectively). Root DW was more variable among genotypes than was shoot DW (Table 3); there was high intraspecific variation among white lupins. The CVs increased among lupin genotypes when including blue lupin and yellow lupin, especially for the root:shoot ratio and root DW (CV difference > 10 %).

**Table 3.** Descriptive statistics of traits and growth parameters of 12 lupin genotypes grown for 33 days as sole plants in rhizotrons (Expt. 1). The mean data for each genotype (*n* = 3–5), mean across genotypes, CV and *P* values of comparisons between genotypes are given for each variable. Minimum and maximum values are underlined with a dotted and a full line, respectively. CV appears in bold if ≥30 %. Significant differences are shown in bold (*P* < 0.05). Root vertical expansion rate data were transformed with X^2^, WWL: winter white lupin; SWL: spring white lupin; BL: blue lupin; YL: yellow lupin. Mean genotype data of each parameter (row) with different letters are significantly different (*P* < 0.05).

		WWL-1	WWL-2	WWL-3	WWL-4	WWL-5	WWL-6	WWL-7	WWL-8	SWL-1	SWL-2	BL	YL	Mean	CV among WL (%)	CV overall (%)	*P*
Seed	Seed DW (mg per seed)	208	225	247	250	253	257	277	308	227	248	160	130	233	11	21	−
	Seed N content (mg per seed)	9.9	13.2	12.8	13.4	14.1	13.0	15.8	22.1	13.6	12.0	7.3	8.7	13	23	29	−
Final DW	Shoot DW (mg per plant)	190.8 f	240.8 cde	217.8 def	230.4 de	253.3 bcd	226.1 def	253.8 bcd	307.4 a	271.7 abc	288.8 ab	211.2 ef	123.4 g	234.63	14	21	**<0.0001**
	Root DW (mg per plant)	54.34 d	73.4 bc	67.6 cd	73.7 bc	70.5 cd	72.4 bcd	68.4 cd	90.5 b	74.7 bc	109.6 a	117.9 a	33.6 e	75.54	20	**30**	**<0.0001**
	Nodule DW (mg per plant)	2.34 b	2.94 b	3.22 b	2.99 b	2.47 b	2.65 b	2.42 b	2.81 b	3.26 b	6.85 a	2.46 b	0.38 b	2.90	**41**	**50**	**0.0001**
	Root:shoot ratio	0.297 c	0.317 c	0.327 c	0.334 bc	0.289 c	0.331 bc	0.279 c	0.303 c	0.287 c	0.406 b	0.569 a	0.276 c	0.33	10	24	**<0.0001**
Root system	Root length (cm per plant)	105 d	147 d	135 d	148 d	134 d	150 cd	113 d	195 bc	221 b	348 a	217 b	47 e	163.42	**42**	**46**	**<0.0001**
	Root vertical expansion rate (cm day^−1^)	0.98 f	1.56 bc	1.26 def	1.28 de	1.18 ef	1.32 cde	1.00 f	1.48 bcd	1.63 b	1.90 a	1.54 bc	1.01 ef	1.35	21	21	**<0.0001**
	Root density at 25 DAS (%)	9.87 de	11.47 bcd	13.56 bcd	12.95 bcd	11.17 cd	12.87 bcd	9.87 de	16.29 b	15.1 bc	21.88 a	11.65 bcd	4.29 e	12.58	27	**34**	**<0.0001**

Among white lupins, WWL-1 had the lowest seed DW (208 mg per seed) and seed N content (9.9 mg per seed) and exhibited the lowest values of shoot and root growth and soil exploration (shoot DW: 190.8 mg per plant; root DW: 54.34 mg per plant; root length: 105 cm per plant; root vertical expansion rate: 0.98 cm day^−1^; root density 25 DAS: 9.87 %). When comparing the eight winter white lupins, WWL-8 had the highest seed DW (308 mg per seed) and seed N content (22.1 mg per seed) and exhibited the highest values of shoot and root DW (307.4 and 90.5 mg per plant, respectively), root length (195 cm per plant) and root density at 25 DAS (16.29 %). Among all white lupins, SWL-2 showed the maximum values for all root variables (root DW: 109.6 mg per plant; root length: 348 cm per plant; root vertical expansion rate: 1.90 cm day^−1^; root density at 25 DAS: 21.88 %) in spite of a lower shoot DW than that of WWL-8 (SWL-2: 288.8 mg per plant and WWL-8: 307.4 mg per plant). On the whole, except for root DW being higher for blue lupin (117.9 mg per plant), SWL-2 showed the maximum values among all lupins. For all plant traits, except seed N content and root vertical expansion rate, yellow lupin exhibited the minimum values.

#### Lupin early growth and root exploration.

**Figure 1.**
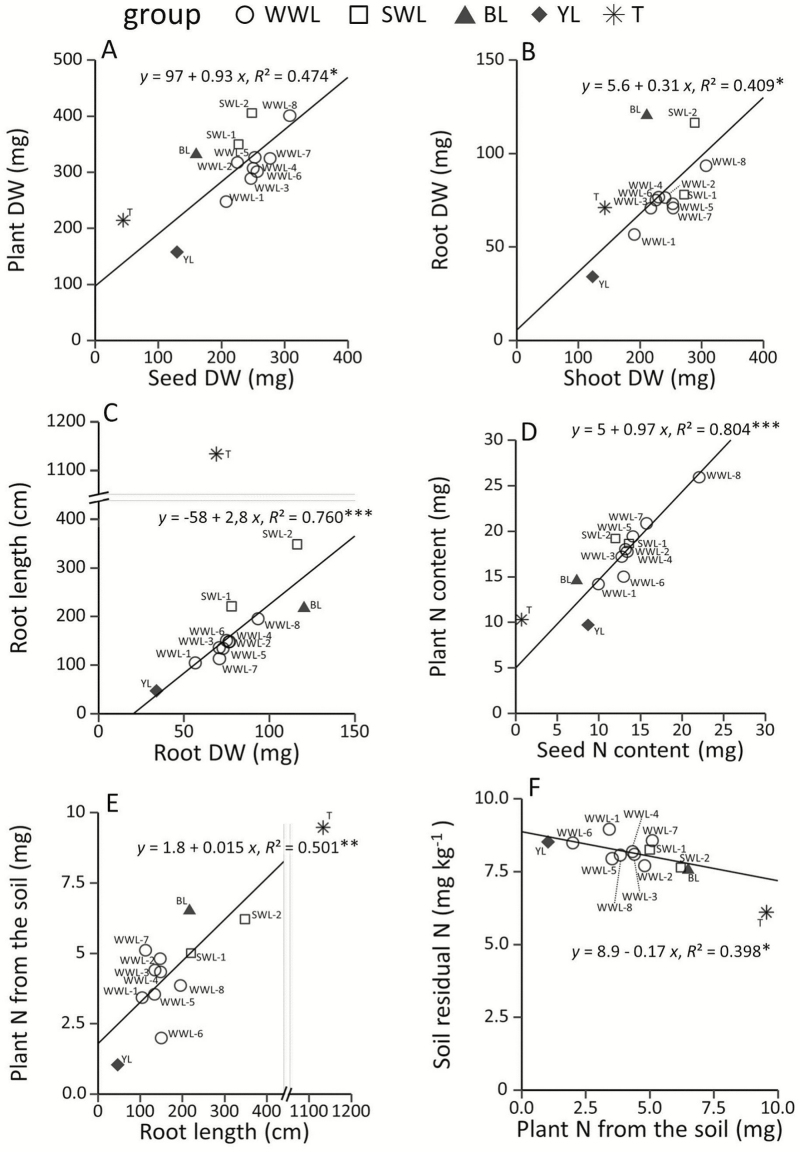
Correlations between the measured variables (A) plant DW and seed DW; (B) root DW and shoot DW; (C) root length and root DW; (D) plant N content and seed N content; (E) plant N from the soil and root length; and (F) soil residual N and plant N from the soil in Expt. 1 for 12 lupins and triticale. WWL: winter white lupin; SWL: spring white lupin; BL: blue lupin; YL: yellow lupin; T: triticale. Variables are expressed per plant or per seed. Regression equations and *R*^2^ are calculated for 12 lupins, not taking into account the triticale data. Correlations are significant at the respective probability levels. *P ≤ 0.05, **P ≤ 0.01, ***P ≤ 0.001.

All lupins exhibited slow growth during the first month, and plant DW was on average only 1.4 times the value of seed DW. A heavier seed resulted in a higher final plant weight (Fig. 1A, linear regression, *F*_1, 10_ = 9.0, *P* = 0.013), although early growth rates were higher for spring white lupins and blue lupin than for winter white lupins. WWL-8 ended up with the same final biomass as SWL-2 in spite of having higher seed reserves.

Root DW and shoot DW were correlated (Fig. 1B, linear regression, *F*_1, 10_ = 6.9, *P* = 0.025), but SWL-2 and blue lupin had higher root:shoot ratios than did the other genotypes. Blue lupin had the highest root DW, which was 3.5 times greater than that of yellow lupin (Table 3). Among white lupins, the root DW of SWL-2 was two times greater than that of WWL-1. Spring white lupins and blue lupins had the longest root systems, which were up to 5.9 times longer than that of yellow lupin. Root length was also highly variable among white lupins (CV = 42 %), and SWL-2 had a root system 3.3 times longer than that of WWL-1.

Genotypes presenting high root DWs generally also presented high root lengths, high root vertical expansion rates and high root densities (Table 3). Figure 1C shows a high correlation between root length and root DW (linear regression, *F*_1, 10_ = 31.7, *P* = 0.0002). However, a higher specific root length was observed for spring white lupins than for winter white lupins (WWL: 1.97 cm mg^−1^ vs. SWL: 3.07 cm mg^−1^). Blue lupin showed the highest root DW but exhibited one of the lowest specific root lengths (1.84 cm mg^−1^).

**Figure 2.**
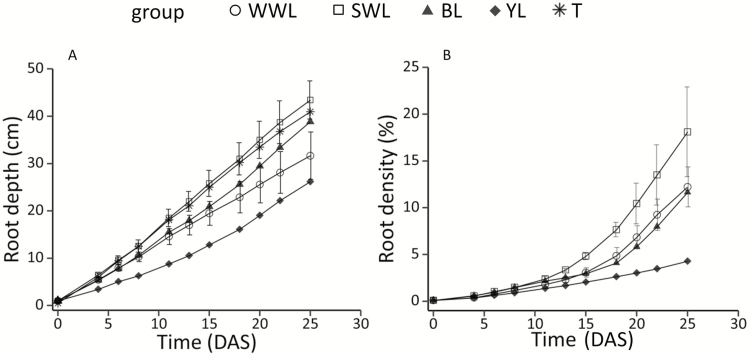
(A) Root vertical expansion for lupins and triticale and (B) root density expansion for lupins during Expt. 1. WWL: winter white lupin; SWL: spring white lupin; BL: blue lupin; YL: yellow lupin; T: triticale. Standard deviations of groups representing several genotypes are displayed.

All groups displayed similar trends of root vertical expansion and density expansion (Fig. 2A and B). Root vertical expansion was linear in the early growth phase and ranged from 1.01 cm day^−1^ (yellow lupin) to 1.75 cm day^−1^ (spring white lupins). Root density started with a lag phase characterized by a low expansion rate until 15 DAS, where the maximal value was 4.8 % (spring white lupins), but the root density increased linearly and more rapidly after 18 DAS, reaching a maximum of 18 % at 25 DAS (spring white lupins).

Yellow lupin exhibited the lowest rooting depth and the slowest root density development. Blue lupin had a higher root vertical expansion rate than did winter white lupins but a similar root density expansion. Spring white lupins had the highest density expansion rate in the linear phase (18–25 DAS) as well as density at 25 DAS. Among white lupins, root density (18–25 DAS) increased by 0.84 % per day (WWL-1) up to 1.95 % per day (SWL-2).

#### Early use of N sources.

**Figure 3.**
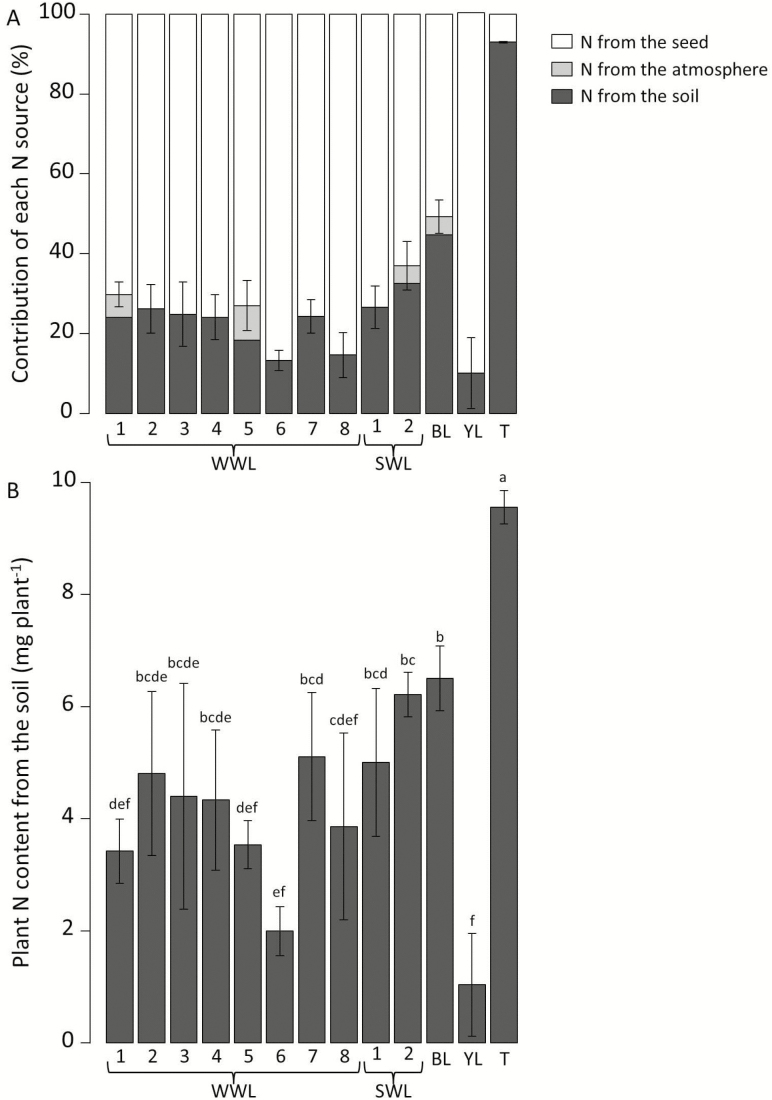
(A) N content according to N sources for 12 lupins and triticale. Error bars represent standard deviations of exogenous N percentage; (B) plant N content originating from the soil, ANOVA: *F*_12, 47_ = 14.6, *P* < 0.0001. WWL: winter white lupin; SWL: spring white lupin; BL: blue lupin; YL: yellow lupin; T: triticale. Different letters indicate significant differences (*P* < 0.05) as shown by the Tukey’s test.

At the end of the experiment, N content varied from 9.71 to 25.91 mg per plant among lupin genotypes (Fig. 1D). On the whole, lupin relied mostly on seed N for the first month of growth (Fig. 3A), and plant N reflected differences in seed N (Fig. 1D, linear regression, *F*_1, 10_ = 40.9, *P* < 0.0001). However, among white lupins, exogenous N proportion ranged from 13 to 37 %. The exogenous N proportion was 10 % in yellow lupin and 49 % in blue lupin (Fig. 3A).

All genotypes initiated nodules during the experimental period (33 days). SWL-2 exhibited a higher nodule DW than all the other white lupin genotypes (Table 3). Only WWL-1, WWL-5 and SWL-2 as well as blue lupin started fixing a measurable amount of N_2_ during the experiment. For the other genotypes, the N content of plants grown in N-free sand did not exceed the amount already present in the seed (data not shown), and the quantity of N from symbiotic fixation is assumed to be 0. The %Ndfa values calculated for total N were 8.6, 5.7, 4.6 and 4.5% for WWL-5, WWL-1, blue lupin and SWL-2, respectively (Fig. 3A).

Lupins absorbed a low quantity of N from the soil (mean = 4.2 mg per plant, max = 6.5 mg per plant) (Fig. 3B). However, plant N absorbed from the soil was correlated with root length (Fig. 1E, linear regression, *F*_1, 10_ = 10.1, *P* = 0.010) as well as with root DW, root density and root vertical expansion rate. Genotypes that started fixing N_2_ (WWL-5, WWL-1, blue lupin and SWL-2) did not show a lower mineral soil N uptake.

There were limited effects of lupin growth on soil residual N. No differences between lupin genotypes and no significant differences with the bare soil control (residual N: 8.15 ± 0.58 mg kg^−1^) were observed. However, there was a significant effect of plant mineral soil N uptake on soil residual N (Fig. 1F, linear regression, *F*_1, 10_ = 6.611, *P* = 0.027).

### Contrasting early growth and N use strategies of lupin and triticale in pure and mixed treatments in the first month

#### Lupin and triticale in pure treatments in the first month (Expt. 1).

Both winter white lupins and spring white lupins have a seed DW that is more than five times higher than that of triticale (Table 4). On average, one lupin seed contains up to 20 times more N than one triticale seed. In Expt. 1, the shoot DW of white lupins was higher than that of triticale. After 1 month of growth, triticale plant DW was 4.8 times more than the seed DW (this ratio was 1.4 for lupins). After 1 month of growth, white lupins and triticale had a similar root DW (Table 4). The difference in total plant DW between white lupins and triticale after 1 month was smaller than the difference in seed DW at the start of the experiment, indicating that triticale had 16.1 and 6.7 times faster growth than winter white lupins and spring white lupins, respectively (Fig. 1A). The root:shoot ratio was lower in white lupins (0.309 and 0.340 in winter white lupins and spring white lupins, respectively) than in triticale (0.500). Root length of triticale represented 8.0 and 4.1 times that of winter white lupins and spring white lupins, respectively. As shown in Fig. 1C, the specific root length of triticale amounted to 16 cm mg^−1^, which was much higher than that of winter white lupins and spring white lupins (2.0 and 3.1 cm mg^−1^, respectively). The root vertical expansion rate of triticale (1.67 cm day^−1^) was higher than that of winter white lupins (1.26 cm day^−1^) but similar to that of spring white lupins (1.75 cm day^−1^). Although starting with a seed N content ~19 times higher than in triticale, white lupins contained only 1.8 times more N than did triticale in the final plant. Triticale accumulated 93 % of its final N content during growth, whereas white lupin N content still depended on seed N for 77 % (winter white lupins) and 68 % (spring white lupins) (Fig. 3A). With a similar root mass but higher root length, triticale was able to absorb more mineral soil N than were white lupins (Fig. 1E), which resulted in a lower quantity of residual mineral N in the soil (Fig. 1F).

**Table 4.** Descriptive statistics of traits and growth parameters of triticale in comparison with winter white lupin and spring white lupin grown for 33 days as sole plants in rhizotrons (Expt. 1). The mean data for each group (T: *n* = 5; SWL: *n* = 9; WWL: *n* = 38) and *P* values of comparison between triticale and each lupin group are given for each variable. Significant values are shown in bold (*P* < 0.05). For comparison WWL-T, root length data were transformed with log(1 + X). WWL: winter white lupin; SWL: spring white lupin; T: triticale.

		WWL	T	SWL	*P*	Comparison T-SWL
					Comparison T-WWL	
Seed	Seed DW (mg per seed)	253	45	237	−	−
	Seed N content (mg per seed)	14.4	0.7	12.9	−	−
Final DW	Shoot DW (mg per plant)	240.8	143.3	279.3	**<0.0001**	**<0.0001**
	Root DW (mg per plant)	71.30	70.92	90.18	0.9456	0.0629
	Root:shoot ratio	0.309	0.500	0.340	**0.0053**	**0.0022**
Root system	Root length (cm per plant)	141	1133	277	**0.0003**	**0.0042**
	Root vertical exp. rate (cm day^−1^)	1.26	1.67	1.75	**0.0004**	0.3933

#### Lupin and triticale in mixed compared to pure treatments in the first month (Expt. 2).

The mixtures showed intermediate values between pure treatments for shoot and root dry matter accumulation and for N acquisition (Table 5). Lupin represented 67.4 % of shoot biomass of the mixture. Root traits and strategy of N acquisition of lupin and triticale were not significantly different between the pure and mixed treatments (Table 6). After 1 month of growth in mixed pairs, the presence of triticale as a neighbour plant did not affect the differences between the four most-contrasting lupin genotypes identified in Expt. 1 (WWL-1, WWL-8, SWL-1, SWL-2; interaction between the factors ‘lupin genotype’ and ‘pure or mixed treatment’: *F*_3, 32_ = 0.1991, *P* = 0.896). In the first month, there were almost no interactions between plants, as shown by the Cb values that were very close to 0 and the RYT values close to 1 regardless of traits. The contrasting strategies of lupin and triticale towards early growth and N use observed in pure treatments were preserved in mixed treatments (Table 6). Total plant N content (lupin + triticale) from the soil was higher in mixed treatments than in pure lupin (Table 5), and lupin contributed to only 24.5 % of that value in the mixture, indicating that triticale allowed for higher mineral soil N uptake by the mixture over pure lupin without limiting lupin early growth.

**Table 5.** Performances of two-plant stands consisting of white lupin and triticale grown in rhizotrons. The mean data for each group (*n* = 3–20), means across groups and *P* values of comparisons between pure lupin, pure triticale and mixed stands are given for each variable. Lupin % represents the proportion of lupin in the mixed stands for each variable. In Expt. 2, soil residual N data were transformed with X^1.5^; in Expt. 3, soil residual N was transformed with X^−0.5^. Group mean data of each parameter (row) with different letters are significantly different within each treatment (*P* < 0.05). Significant values are shown in bold (*P* < 0.05).

	Expt. 2 (30 days)				Expt. 3 (58 days)			
	Pure lupin	Pure triticale	Mixed stand	*P*	Pure lupin	Pure triticale	Mixed stand	*P*
			Lupin %				Lupin %	
Shoot DW (mg per 2 plants)	626 a	325 c	453 b	**<0.0001**	7880 a	5214 b	8364 a	**<0.0001**
			67.4				42.0	
Root (+ nodule) DW (mg per 2 plants)	174 a	103 b	134 b	**<0.0001**	3002 b	3534 ab	3990 a	**0.0036**
			54.7				31.9	
Plant N content (mg per 2 plants)	43.16 a	24.02 b	30.75 b	**<0.0001**	365.72 a	81.81 c	260.51 b	**<0.0001**
			65.7				71.5	
Exogenous N content (mg per 2 plants)	10.49 c	22.63 a	13.81 b	**0.0105**	327.67 a	80.52 c	240.84 b	**<0.0001**
			25.6				69.5	
Plant N content from soil (mg per 2 plants)	8.08 c	22.63 a	13.53 b	**<0.0001**	71.41 b	80.52 a	86.76 a	**0.0011**
			24.5				15.2	
Soil residual N (mg kg^−1^)	11.3 a	8.2 b	10.6 a	**<0.0001**	0.31 a	0.32 a	0.27 a	0.4492

**Table 6.** Comparison of white lupin and triticale grown as pure stands and mixed stands for 30 days in rhizotrons (Expt. 2). For lupin and triticale, the mean data for each group (*n* = 3–20), *P* values of comparisons between pure and mixed stands, competitive balance index and relative yield total are given for each variable.

	White lupin			Triticale			Competitive balance index	Relative yield total
	Pure	In mixed stand	*P*	Pure	In mixed stand	*P*		
Seed DW (mg per seed)	272.95		−	39		−	−	−
Seed N content (mg per seed)	16.3		−	0.7		−	−	−
Shoot DW (mg per plant)	312.9	305.1	0.4912	162.4	147.5	0.3951	0.07	0.94
Root DW (mg per plant)	81.45	81.15	0.9455	51.73	46.87	0.5760	0.10	0.95
Nodule DW (mg per plant)	5.42	5.62	0.6977	−	−	NA	−	−
Root:shoot ratio	0.276	0.285	0.3921	0.291	0.320	0.4944	−0.06	0.95
Root length (mg per plant)	282	268	0.5136	874	876	0.9948	−0.05	0.98
Root vertical expansion rate (cm day^−1^)	2.22	2.19	0.7602	2.00	1.84	0.2108	0.07	0.95
Plant N content (mg per plant)	21.58	20.16	0.1526	12.01	10.59	0.3854	0.06	0.91
Exogenous N content (mg per plant)	5.24	3.91	0.1549	11.32	9.90	0.3853	−0.16	0.81
Plant N content from soil (mg per plant)	4.04	3.64	0.6211	11.32	9.90	0.3853	0.07	0.89

### Contrasting growth, N use strategies, and competitive ability of lupin and triticale after 2 months in pure and mixed treatments

#### Lupin and triticale in pure treatments after 2 months (Expt. 3).

After 58 days, white lupin (WWL-6) shoot DW was higher than that of triticale, but triticale had produced higher root DW than did lupin (Table 5). Lupin had a higher N content than triticale, but its strategy towards N use had changed compared with the strategy observed in the first month (Expt. 2). Lupin N content coming from the seed was only 10 %; 69.8 % of lupin total N came from the atmosphere. At this stage (after 2 months), triticale absorbed only slightly more N from the soil than did lupin (13 % more, in contrast to 180 % more after 1 month in Expt. 2).

#### Interactions and complementarity in mixed treatments after 2 months (Expt. 3).

The strategy of N acquisition of lupin was strongly modified in mixed treatments compared to that of pure treatments. Exogenous N content in lupins remained the same regardless of neighbouring plant. Lupin took up significantly less N from the soil and fixed more N when grown with triticale compared with the pure treatments, which translated into a higher %Ndfa in the mixture (Table 7, 82.7 % in mixed stand vs. 69.8 % in pure stand). The acquisition of mineral soil N per plant was reduced in mixture compared to pure treatments.

**Table 7.** Comparison of white lupin and triticale grown in pure stands and in mixed stands for 58 days in rhizotrons (Expt. 3). For lupin and for triticale, the mean data for each group (*n* = 5), *P* values of comparison between pure and mixed stands, competitive balance index and relative yield total are given for each variable. Significant differences are shown in bold (*P* < 0.05).

	White Lupin			Triticale			Competitive balance index	Relative yield total
	Pure	In mixed stand	*P*	Pure	In mixed stand	*P*		
Seed DW (mg per seed)	280.23		−	37.27		−	−	
Seed N content (mg per seed)	19.0		−	0.6		−	−	
Shoot DW (mg per plant)	3940	3510	0.0903	2608	4854	**<0.0001**	−0.74	1.38
Root DW (mg per plant)	1277	1197	0.1820	1767	2558	**0.0013**	−0.43	1.19
Nodule DW (mg per plant)	223.3	234.0	0.2510	−	−	−	−	−
Root:shoot ratio	0.382	0.410	0.1453	0.682	0.530	**0.0473**	0.32	−
Plant N content (mg per plant)	182.86	186.30	0.7487	40.9	74.22	**<0.0001**	−0.58	1.42
Exogenous N content (mg per plant)	163.84	167.28	0.7483	40.24	73.58	**<0.0001**	−0.58	1.42
Plant N content from soil (mg per plant)	35.71	13.20	**<0.0001**	40.24	73.58	**<0.0001**	−1.60	1.10
Plant N content from N_2_ fixation (mg per plant)	128.13	154.08	**0.0331**	−	−	−	−	−
%Ndfa	69.8	82.7	**<0.0001**	−	−	−	−	−

After 2 months, triticale mixed with lupin showed higher values of shoot and root DW and mineral soil N uptake per plant than when grown in pure treatments (Table 7). Negative Cb values showed that triticale benefited from the interactions in mixture with lupin (Table 7). The RYT values were higher than 1, indicating a higher shoot biomass (+38 %), root biomass (+19 %), soil N uptake (+10 %) and plant N content (+42 %) in mixed treatments compared with pure treatments. In pure treatments, triticale shoot DW was lower than that of lupin, but it yielded almost twice as much when grown with lupin. Moreover, triticale grown with lupin absorbed 74 mg N from the soil but only 40 mg when in competition with another triticale plant. The enhanced growth of triticale in mixed treatments also strongly increased the contrast of root DW between lupin and triticale. After 2 months, the increase in triticale root DW in mixed treatments allowed for higher root DW in the mixture than in pure lupin (Table 5) as well as a higher total DW (*P <* 0.0001, data not shown). The highest total plant N content was in pure lupin, with 10 % coming from the seed (Table 5). The mixture and pure triticale absorbed significantly more N from the soil than did pure lupin, but soil residual N was depleted from the initial level regardless of treatment (Table 5).

## Discussion

Our results show that there are differences among lupin species and between genotypes of one species for traits related to early growth and mineral soil N uptake. Even so, all lupins demonstrated slow early growth and low ability to absorb mineral soil N compared to triticale. However, in lupin–triticale mixtures, the strong contrast in early growth strategies between species results in a higher competitive ability for soil mineral N of the mixture in comparison with pure lupin. Our results demonstrate that complementarity for N resource use in mixtures of cereal–grain legumes can appear early in the growth cycle.

### Inter- and intraspecific variation among lupin genotypes

Although all lupins displayed a low soil N uptake ability during early growth, there were differences among winter white lupins for traits related to the root system and N use after 1 month of growth. The inclusion of different cultivars and species of lupins (spring white lupins, blue lupin and yellow lupin) increased the level of variation. Other studies have examined intraspecific variability within lupin species. In a semi-hydroponic system, 10 *L. angustifolius* genotypes (narrow-leafed lupin) selected from a large germplasm pool for maximizing differences (1301 genotypes comprising landraces and wild accessions) showed a CV of 36 % for root DW (Chen *et al.* 2011). In experimental conditions similar to our study, nine contrasting legume species showed a CV of 82 % for root DW (Dayoub *et al.* 2017). In our study, root length had a CV of 42 % among white lupins, which is higher than that (37 %) measured by Chen *et al.* (2011) in spite of the presumably narrower genetic base in our study. This confirms that there is high variation for root traits in the pool of white lupins under selection. Among lupins, we observed higher variation for root DW than for shoot DW, which is consistent with previous studies (Chen *et al.* 2011; Dayoub *et al.* 2017). Below-ground variation is usually less studied than above-ground variation and may thus often be underestimated.

The level of lupin shoot DW and root DW measured in our three experiments is in agreement with that measured by Tang *et al.* (1999) in a 6-week experiment. The rate of root vertical expansion was within the range described in Dayoub *et al.* (2017) for nine contrasting legume species. Root density showed the same trend as that of pea described by Thorup-Kristensen (1998), with a somewhat longer lag phase and a higher increase in root density growth rate after 15 DAS.

Many early growth variables were correlated (Fig. 1). Correlations between plant DW and seed DW (Fig. 1A) and plant N content and seed N content (Fig. 1D) may have largely been a function of the limited increases in DW and plant N during the first month of growth by lupin. Root:shoot ratios of the lupins exhibited a narrow range of variation with the exceptions of narrow-leafed lupin and SWL-2, which had notably higher root DW and root:shoot ratio. Root:shoot ratio is allometric and changes with size (Müller *et al.* 2000); during the early stages, this ratio is small but increases with plant age. Both triticale and lupin had higher root:shoot ratio after 2 months (Expt. 3) than after 1 month (Expt. 2).

Among winter white lupins, there were consistent differences for all variables between the genotype exhibiting the highest values and the one exhibiting the lowest: WWL-8 was the quickest winter white lupin to establish a dense, deep and long root system and had 1.7 times more root DW than did WWL-1, which had the slowest start. Winter white lupins seemed to have a slower early growth rate than did spring white lupins, which may be a consequence of breeding for frost tolerance with a slower leaf development and a delayed floral initiation in winter types. Among the tested lupin genotypes, SWL-2 showed the strongest ability to develop a dense, deep and long root system.

Yellow lupin has relatively light seeds containing little N, yet this species showed a low use of exogenous N. This may have resulted from a slow start, its low N requirement and its very low root system expansion. Blue lupin also has relatively light seeds, but this species showed a high exogenous N proportion. Blue lupin did not have high shoot DW but did exhibit high values for root traits (root DW, root:shoot ratio, root vertical expansion rate) and N uptake ability. Blue lupin produces less root length than does white lupin for a similar root DW in accordance with Clements *et al.* (1993).

Comparison between lupin genotypes and species for N use showed that root DW, root length, root depth expansion rate and root density (correlated variables) have an impact on early mineral soil N uptake. In a simulation study for a range of crops (e.g. wheat, barley, lupin, soybean), Dunbabin (2007) found that variables controlling the spatial intensity of foraging (here, root density) and rate of root system establishment (here, root depth expansion rate) were the most important for N uptake in competition with weeds.

### Contrasting strategies between lupin and triticale for early growth and N use

Triticale absorbed more mineral soil N than did lupin, as early as 1 month after sowing. Numerous studies have shown that cereals can absorb more mineral soil N than can legumes (Martin and Snaydon 1982; Jensen 1996; Hauggaard-Nielsen *et al.* 2001; Ghaley *et al.* 2005; Corre-Hellou *et al.* 2007), but this has rarely been demonstrated in early growth stages nor has it been linked to seed N and root traits. Lupin seeds contain a high amount of N and contribute, on average, 75 % of plant N for N for seedling growth in the first month. Dayoub *et al.* (2017) found among legumes a range of reliance on seed N from 6 % in *Medicago sativa* to 77 % in *Arachis hypogea* during early growth (35 days after germination). Soybean (*Glycine max*) also had a high N content and concentration like lupins in our experiment, but it relied on seed N for only 49 % of plant N (Dayoub *et al.* 2017). Lupin thus seems to be among the legume species that relies the most on seed N in the first month of growth. This may be largely a function of the relatively large seed N reserves and slow early growth (in comparison to other legumes) of lupin. Triticale seedlings grew faster and relied on seed N for only 7 % of plant N after 1 month. Although N uptake varied between lupins, none of the varieties examined significantly reduced soil mineral N, due to the low N uptake of all the lupin varieties examined. Triticale seedlings, which started with 18 times less N in the seed, absorbed significantly more soil mineral N.

Triticale has a distinctly different root system than does lupin. The finer roots of triticale show a lower resource investment per unit of root length, with the capacity to produce seven times more root length than lupin can with the same root mass. Martin and Snaydon (1982) also noted that the ability of cereals to produce finer roots than legumes was responsible for their higher below-ground competitive ability. Triticale produced much more root length than did lupins and had explored all of the rhizotron soil volume at the end of the 1-month experimental period, whereas lupin had not. In comparison to lupin, triticale had lower seed DW, much lower seed N content, higher early growth rate, finer roots and a higher capacity to produce a dense root system.

### Mixing triticale with lupin allows better performance without negatively impacting lupin growth

The strong contrasts between lupin and triticale are maintained when the two species are grown together for 1 month. In Expt. 2, we detected no impact of ‘neighbour identity’ on the plants, as there was nearly no interaction between the plants during the first month of growth in our setting. Lupin and triticale were both allowed to access soil resources and light in the same way regardless of the neighbouring plant. Plants with a low biomass have a low competitive ability at this early stage. Thus, there was also no impact of lupin genotype choice on triticale and on performances of the mixed stands. Because of the higher mineral soil N uptake of triticale in comparison with lupin, mixed species pairs had greater N uptake than pure lupin, but lower N uptake than pure triticale after 1 month of growth. In our 1-month experiments, all lupin genotypes initiated nodules, but N_2_ fixation could be detected in only four of these genotypes. The beginning of N_2_ fixation was not critical for N content because lupins relied mainly on seed N in the first month.

At the end of the 2-month growth period, plants had started to interact for resources in the rhizotron. The most significant effect of mixing species was the better growth of triticale when grown with lupin than with a conspecific plant. N was limiting in Expt. 3 and, in pure triticale, each triticale plant had to share N with another triticale plant with a strong ability to absorb N. When growing with a lupin plant instead of a conspecific plant, the triticale plant had access to more N because of the lower N uptake ability of lupin. In contrast, lupin growth was not significantly affected by growing with a triticale plant instead of a conspecific. Lupin DW, root:shoot ratio and N content were the same as those in pure lupin, although there may have been a slight decrease (non-significant) of lupin shoot DW caused by the initial competition from triticale. Lupin was apparently affected by triticale mineral soil N uptake, but this did not translate into a lower plant N content because lupin compensated for lower mineral soil N uptake with increased N_2_ fixation. This ability of legumes to change their N acquisition strategy towards more N_2_ fixation when the supply of mineral soil N is limited by uptake from a cereal plant has been reported often (e.g. Jensen 1996; Corre-Hellou *et al.* 2006; Naudin *et al.* 2010). Our study provides additional evidence that this mechanism can occur even before stem elongation of a winter legume, i.e. early in the crop cycle. The switch to more N_2_ fixation by lupin and the enhanced growth and mineral soil N uptake ability of triticale were responsible for increased contrast between lupin and triticale for mineral soil N uptake in mixtures. In our study, the observation of contrasting plant traits of lupin and triticale and RYT values show that the existing complementarity in the below-ground resource capture strategies is enhanced by interspecific competition because of the increase in niche differentiation. Higher productivity of cereal–legume intercrops over sole crops often results from complementarity for N (Jensen 1996; Hauggaard-Nielsen *et al.* 2009; Bedoussac and Justes 2010b; Pelzer *et al.* 2012; Bedoussac *et al.* 2015), but it has rarely been demonstrated that this complementarity could appear early in the cropping cycle.

In Expt. 3, nearly all mineral soil N was used by the plants within the duration of the experiment, indicating that mineral soil N uptake and growth were limited by N availability, which may have strengthened lupin N_2_ fixation and limited triticale growth. The differences between pure lupin and pure triticale and between pure lupin and mixed treatments regarding plant N uptake and soil residual N were thus lower than expected from our hypotheses and from Expt. 2. It is likely that both pure triticale and mixed treatments had depleted their mineral soil N earlier than did pure lupin.

### Potential applications and perspectives

No white lupin showed strong potential for early mineral soil N uptake. With less seed N, blue lupin took up more mineral soil N. However, selecting white lupin with small seeds is unlikely to increase early mineral soil N uptake: among winter white lupins, the smallest-seeded genotype (WWL-1) had slow early growth. This genotype did not absorb more mineral soil N than did WWL-8, which had 2.2 times more seed N. It may be difficult to breed for winter white lupins with a low seed N content and a fast early root system because it seems that those characteristics are correlated. Moreover, thousand grain weight and grain protein concentration are key criteria for end-users and the potential of breeding white lupin for higher competitive ability may be restricted by the shallow genetic basis currently used in breeding programmes.

Our study focused on early growth and underlined some mechanisms impacting the competitive ability for N in lupin and triticale in pure and mixed species pairs. These mechanisms will have an impact on the competitive ability of each plant later in the cycle. Zhang and Lamb (2012) found that early differences between species for competitive effect did not vary substantially with plant age. As suggested by Fayaud *et al.* (2014), a better understanding of early growth in intercrop systems could help to adapt management practices to attain specific objectives, such as proper proportion of each species in terms of grain yield. Nevertheless, other mechanisms, such as competition for light and differing phenologies, may in turn alter the competitive balance of plants and of the intercrop system.

This study did not evaluate the potential for above-ground competition which could also compromise lupin growth. In our experiments, it is rather unlikely that competition for light played a role because the plants of each pair were sown at a distance likely preventing them from competing for light. However, in field conditions over a whole cropping season, competition for light can play a role in interactions between intercropped species and weeds. Blue lupin is known to show low ability to provide ground cover (Boundy *et al.* 1982) and is probably not more competitive than white lupin. Intercropping cereals with lupin could also provide benefits in terms of ground cover because it is likely that cereals and lupin show contrasting above-ground architectures and light interception dynamics and, thus, complementarity in the use of light, as has been shown for other legumes (Keating and Carberry 1993; Bedoussac and Justes 2010a).

There is an increasing body of research advocating for increasing crop stand trait diversity to increase crop performance and resilience, including competitive ability against weeds (Malézieux *et al.* 2009; Pakeman *et al.* 2015). Our study of early growth stages in lupin and triticale with respect to growth and N use suggests that intercropping with a cereal can increase complementarity between plants in the crop stand and reduce inter-plant competition compared to plants of the same species. We therefore conclude that lupin–cereal intercropping could help control early weed growth because, at appropriate sowing densities, intercropping could leave less soil mineral N for the weeds compared with lupin sole cropping, without compromising lupin growth.

## Conclusions

From our study, it appears that in spite of the intra-genus differences, the potential for increasing the early competitive ability of lupin by selecting for fast-growing genotypes proficient in accessing soil N may be low. Complementarity between lupin and triticale grown in mixture appears early and is reinforced throughout early growth, allowing low competition between mixed species and higher pressure on soil mineral N than in pure lupin. Therefore, intercropping with a cereal may be a promising option when aiming to grow lupin with a stronger competitive ability against weeds.

## Conflict of Interest

None declared.

## Sources of Funding

This work was supported by Europe and Regional Council of Brittany (France) within the project PROGRAILIVE carried out by the association Pole Agronomique de l’Ouest.

## Contributions by the Authors

All authors designed the experiments and made substantial contributions to the manuscript. R.B. conducted Expt. 3. N.C. conducted Expt. 1, Expt. 2 and analyses.
